# *Pseudomonas syringae* pv. syringae B728a Regulates Multiple Stages of Plant Colonization via the Bacteriophytochrome BphP1

**DOI:** 10.1128/mBio.01178-17

**Published:** 2017-10-24

**Authors:** Regina McGrane, Gwyn A. Beattie

**Affiliations:** Department of Plant Pathology and Microbiology, Iowa State University, Ames, Iowa, USA; University of Toronto

**Keywords:** bacteriophytochrome, epiphytic bacteria, photosensory protein, phyllosphere, phyllosphere-inhabiting microbes, *Pseudomonas syringae*, spermosphere, swarming motility, histidine kinase

## Abstract

Light may be an important environmental signal for plant-associated bacteria, particularly those that live on leaves. An integrated network of red/far-red- and blue-light-responsive photosensory proteins is known to inhibit swarming motility in the foliar plant pathogen *Pseudomonas syringae* pv. syringae B728a. Here we elucidated factors in the red/far-red-light-sensing bacteriophytochrome BphP1 signal transduction pathway and report evidence for a role of BphP1 in multiple stages of the *P*. *syringae* B728a life cycle. We report that BphP1 signaling involves the downstream regulator Bsi (bacteriophytochrome-regulated swarming inhibitor) and an acyl-homoserine lactone (AHL) signal. Loss of *bphP1* or *bsi* resulted in the early initiation of swarm tendrils during swarming motility, a phenotype that was dependent on red/far-red light and reversed by exogenous AHL, illustrating that the BphP1-Bsi-AHL pathway inhibits the transition from a sessile state to a motile state. Loss of *bphP1* or *bsi* resulted in larger water-soaked lesions induced on bean (*Phaseolus vulgaris*) pods and enhanced movement from soil and buried plant tissues to seeds, demonstrating that BphP1 and Bsi negatively regulate virulence and bacterial movement through soil to seeds. Moreover, BphP1, but not Bsi, contributed to leaf colonization; loss of *bphP1* reduced survival on leaves immediately following inoculation but enhanced the size of the subsequently established populations. Neither Bsi nor Smp, a swarm motility-promoting regulator identified here, affected leaf colonization, indicating that BphP1-mediated contributions to leaf colonization are, at least in part, independent of swarming motility. These results demonstrate that *P*. *syringae* B728a red-light sensing involves a multicomponent, branched regulatory pathway that affects several stages of its life cycle.

## IMPORTANCE

Microbes on plants are particularly well positioned to exploit light cues based on the importance of light to plant growth. Photosensory proteins enable organisms to sense light and respond to light, but their roles in the life cycles of plant microbes are poorly understood. This study investigated the cellular components and ecological roles of red/far-red-light sensing in the foliar bacterial pathogen *Pseudomonas syringae*. The study demonstrated that a bacteriophytochrome photosensory protein functions via a multicomponent, branched regulatory pathway that operates primarily through red/far-red-light-mediated inhibition. This pathway negatively regulates the transition from sessile to motile states under conditions conducive to swarming motility. It also negatively regulates virulence on bean pods, movement through soil to seeds, and survival following inoculation on leaves, but it positively contributes to the eventual establishment of leaf-borne populations. These results provide strong evidence that light sensing modulates behaviors at multiple stages in the life cycle of a nonphotosynthetic, plant microbe.

## INTRODUCTION

Microbes in many environments have proteins that enable the perception of light. Given that light capture is critical to plant growth, microbes on plants are particularly well positioned to exploit light cues. Microbes on aerial leaf surfaces may encounter a full spectrum of solar radiation, whereas those within plant tissues encounter light that has been modulated in intensity and quality via absorption by pigments and scattering as it passes through cell walls and intercellular air spaces (1, 2). The absorption of blue and red light, but not far-red light, by chlorophyll, carotenoids, and other pigments (3) increases the availability of far-red light over red and blue light within plant tissues. Photoreceptors are proteins with a photosensory domain that is activated when an associated chromophore is excited by a specific wavelength of light (4); these proteins enable cells to sense light. Photoreceptors that are widely distributed among plant-associated bacteria and fungi include phytochromes and light-oxygen-voltage (LOV) domain-containing proteins (5, 6). Phytochromes respond to red and far-red light, whereas LOV proteins respond to blue light. Despite the potentially greater importance of far-red light, and thus phytochromes, to plant-associated bacteria, LOV proteins have been more extensively examined for their role in the ecology of these bacteria.

Following the identification of phytochromes in plants and cyanobacteria, bacteriophytochromes were first discovered as light-regulated histidine kinases in *Deinococcus radiodurans* and *Pseudomonas aeruginosa* (7). Biochemical studies with bacteriophytochromes of *D. radiodurans* and the foliar pathogen *Pseudomonas syringae* pv. tomato strain DC3000 demonstrated that these proteins associate with a biliverdin chromophore (8) compared to the phytocyanobilin chromophore typical of cyanobacterial phytochromes. Bacteriophytochromes have been found to regulate pigment production in *D. radiodurans* (7) and heat tolerance and pyocanin production in *P. aeruginosa* (9). In stem-nodulating *Bradyrhizobium* strains, bacteriophytochromes regulate a metabolic shift from chemoheterotrophy when cells are in soil to photoheterotrophy when cells are in stem nodules, thus allowing the cells to exploit a more energetically favorable form of metabolism when light is available (10, 11). Although the bacteriophytochromes of *Xanthomonas* species lack the histidine kinase domain characteristic of those in other plant bacteria (12), these bacteriophytochromes were recently shown to suppress light-mediated phenotypes and contribute to the virulence of *Xanthomonas campestris* pv. campestris on *Arabidopsis thaliana* (12), thus potentially minimizing virulence trait expression to avoid light-enhanced plant defenses (13, 14). Histidine kinase-based bacteriophytochromes contribute to light-mediated suppression of conjugation in the tumor-inducing pathogen *Agrobacterium fabrum* (15) and tolerance to oxidative stress generated by singlet oxygen in the root-colonizing species *Azospirillum brasilense* (16). They also contribute to the light-mediated suppression of swarming motility in *P. syringae* pv. syringae B728a (17) and potentially to the light-mediated repression of swarming motility (18) and growth in leaves (19) of *P. syringae* pv. tomato DC3000.

*Pseudomonas syringae* is a species complex that causes diseases on a wide range of economically important crops (20). *P. syringae* is commonly found on plant surfaces in the phyllosphere, but it is also found in clouds, waterways, and snowpack (21). In many of these habitats, including leaf surfaces, *P. syringae* is exposed to light as well as environmental challenges such as oxidative and osmotic stress, suggesting the possibility that light could signal responses to co-occurring stresses. All of the *P. syringae* strains with complete genome sequences thus far have genes encoding a LOV protein and a bacteriophytochrome, with the majority also encoding a second bacteriophytochrome. We have previously demonstrated functions for the LOV protein and one of the two bacteriophytochromes, BphP1, in *P. syringae* pv. syringae B728a (17). These studies demonstrated that BphP1 responds, directly or indirectly, to red, far-red, and blue light and integrates its response with LOV to regulate swarming motility (17). Swarming motility in *P. syringae* is a coordinated movement requiring biosurfactant production (22) and functional flagella (23). Swarming motility in pseudomonads is influenced not only by light but also by flagellar glycosylation (24–26), reconfiguration of the stators in the flagellar motor (27–29), quorum sensing (30–32), lipopolysaccharide components (33), and high temperature (34). Despite this mechanistic knowledge of swarming motility, our understanding of the regulatory pathway by which it is affected by light is limited.

Here we explore the role of the bacteriophytochrome BphP1 in the photoregulation of swarming motility and other phenotypes in *P. syringae* pv. syringae B728a. BphP1 is composed of an N-terminal chromophore binding domain and a C-terminal histidine kinase domain. Studies with *P. syringae* pv. tomato DC3000 showed that BphP1 is autophosphorylated in response to red light (8), and studies with *P. syringae* pv. syringae B728a showed that the histidine kinase activity of BphP1 is critical for regulation of swarming motility (17). Although following autophosphorylation histidine kinases generally transfer the phosphoryl group to a conserved aspartate in an associated response regulator (RR), BphP1 lacks a clear candidate RR. Rather than being coexpressed with its cognate RR, the *bphP1* gene is cotranscribed with *bphO*, which encodes a heme oxygenase critical for production of biliverdin (8); we designated the heme oxygenase-bacteriophytochrome operon *bphOP1*. One strategy for elucidating the BphP1-mediated signal transduction pathway is to exploit software using a Bayesian network method to predict protein-protein interactions (35), as illustrated by the successful identification of downstream regulatory components of the cyanobacterial histidine kinase NblS (36).

The objectives of this study were to (i) identify the mechanism of BphP1-mediated regulation of swarming motility, (ii) identify downstream components of the BphP1/LOV-mediated signaling pathway, and (iii) characterize the role of BphP1 in the plant colonization and pathogenesis of *P. syringae* pv. syringae B728a. Our findings provide evidence that BphP1 mediates photoregulation of a switch from sessile to active swarming and contributes to movement to seeds in soil, survival and growth on leaves, and lesion development on bean (*Phaseolus vulgaris*) pods. Moreover, BphP1 signaling involves a downstream regulator, designated Bsi (bacteriophytochrome-regulated swarming inhibitor), and an acyl-homoserine lactone (AHL) signal known to repress swarming (32). Collectively, our results illustrate that red/far-red-light sensing in this well-studied pathogen involves a multicomponent, branched regulatory pathway that affects several stages of the *P. syringae* pv. syringae B728a life cycle.

## RESULTS

### BphP1 negatively regulates swarming by repressing the initiation of swarm tendrils.

**FIG 1:**
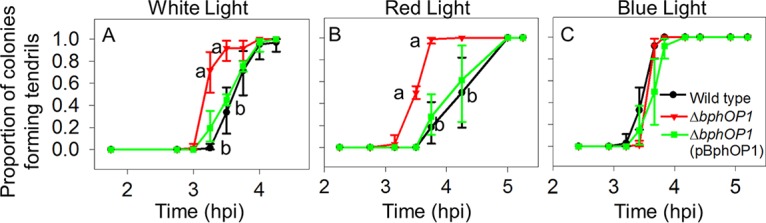
BphP1 regulates swarming initiation and does so through red-light signaling. The proportion of five colonies exhibiting tendril formation was evaluated in the wild-type *P. syringae* pv. syringae B728a, Δ*bphOP1*, and Δ*bphOP1*(pBphOP1) strains in white (A), red (B), and blue (C) light. Values with the same lowercase letter do not differ significantly for comparisons within a single time point (*P* < 0.05 by one-way ANOVA of arcsine-transformed data). Error bars represent the standard errors of the means (SEMs) (*n* = 4). Results are representative of three replicate experiments. hpi, hours postinoculation.

We previously demonstrated that BphP1 negatively regulates swarming motility based on the hyperswarming of *P. syringae* pv. syringae B728a mutants lacking either *bphP1* or the operon containing *bphP1* and the heme oxygenase-encoding *bphO* gene; this regulation does not involve modulating flagellar activity or biosurfactant production (17). In this work, we used the *bphOP1* deletion mutant rather than the *bphP1* deletion mutant to avoid the potential accumulation of biliverdin. We interpret changes in the behavior of the *bphOP1* mutant to reflect BphP1-regulated phenotypes, although elevated levels of the biliverdin precursor heme may have indirect effects. To evaluate whether BphP1 regulation influences swarm tendril initiation, we recorded the time at which a bulge appeared along an otherwise smooth colony edge of the wild-type B728a strain and a Δ*bphOP1* strain during growth on swarm plates. In white light, the Δ*bphOP1* strain initiated tendril formation earlier than the wild type did based on the larger proportion of colonies forming tendrils at 3.25 and 3.5 h postinoculation (hpi) (Fig. 1A). Similar results were observed in red light at 3.5 and 3.75 hpi (Fig. 1B), but not in blue light (Fig. 1C). Expressing the *bphOP1* operon under the control of a constitutive *nptII* promoter in tandem with the native *bphOP1* promoter in the Δ*bphOP1* strain restored the Δ*bphOP1* strain to wild-type behavior (Fig. 1). Thus, BphP1 repressed swarming motility, at least in part, by delaying tendril initiation, and although BphP1 was previously shown to respond to blue and red/far-red light (17), this delay occurred in response to red and white, but not blue, light.

### Of two putative BphP1-interacting proteins, Psyr_2449 (Smp) and Psyr_2699 (Bsi), only Bsi is clearly in the BphP1/LOV regulatory pathway.

**FIG 2:**
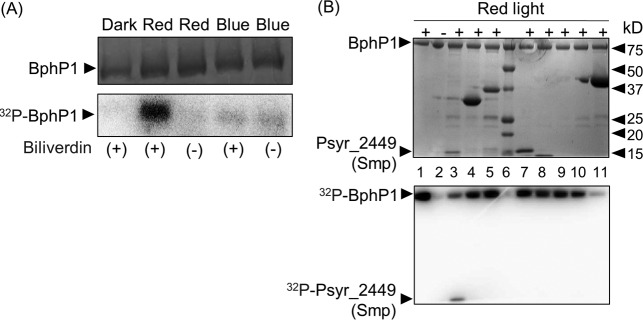
BphP1 autophosphorylates when exposed to red light and can transfer its phosphoryl group to Psyr_2449. (A and B) Following light exposure, purified proteins were subjected to SDS-PAGE (top panels) and exposed to phosphorimaging (bottom panels). (A) Purified His_6_-tagged BphP1 was incubated with [^32^P]ATP in the presence (+) or absence (−) of biliverdin in the dark and in red and blue light. (B) Purified His_6_-tagged proteins were incubated with His_6_-tagged BphP1 and [^32^P]ATP in the presence (+) or absence (−) of red light, as indicated. Lane 1, autophosphorylated BphP1 exposed to red light; lane 2, BphP1 kept in the dark; lane 6, protein standards; and autophosphorylated BphP1 incubated with Psyr_2449 (lane 3), Psyr_4376 (lane 4), Psyr_3433 (lane 5), Psyr_0488 (lane 7), Psyr_0489 (lane 8), Psyr_4392 (lane 9), Psyr_3299 (lane 10), and Psyr_0886 (lane 11). BphP1 autophosphorylation results are representative of at least three independent experiments, and phosphoryl transfer assays were performed for each candidate response regulator a minimum of two times. The positions of protein standards (lane 6) (in kilodaltons) are indicated to the right of the gel.

As a first step to investigate the BphP1 signal transduction pathway in *P. syringae* pv. syringae B728a, we examined BphP1 phosphorylation in response to red (680-nm) and blue (470-nm) light. Purified His_6_-tagged BphP1 exhibited biliverdin-dependent autophosphorylation in response to red light, as was observed previously for BphP1 in *P. syringae* pv. tomato DC3000 (8) (Fig. 2A). However, BphP1 did not show autophosphorylation in response to the blue light provided (Fig. 2A).

We used software designed to predict protein-protein interactions in two-component systems (35) to identify eight candidate BphP1-interacting proteins based on a probability of interaction above 0.001 (Table 1). To evaluate whether the three candidate proteins with the highest probability of interaction, Psyr_2449, Psyr_0886, and Psyr_4376, or two candidate proteins with predicted roles in motility, Psyr_0488 and Psyr_0489, were phosphorylated by BphP1, we expressed each candidate BphP1-interacting protein as a His_6_-tagged derivative, purified it, and coincubated it with His_6_-tagged BphP1 that had been exposed to red light in the presence of [^32^P]ATP (Fig. 2B). Due to the possibility of cross talk between BphP1 and LOV (light-oxygen-voltage) and candidate response regulators, we took a similar approach to identify and purify potential LOV-interacting proteins and tested three of these proteins, Psyr_3433, Psyr_4392, and Psyr_3299, for interactions with BphP1. Out of these eight proteins, only Psyr_2449 was detectably phosphorylated by BphP1 (Fig. 2B). During an extended incubation of Psyr_2449 with autophosphorylated BphP1, radiolabeled BphP1 decreased and radiolabeled Psyr_2449 increased (see Fig. S1A and B in the supplemental material), supporting phosphotransfer activity from BphP1 to Psyr_2449. Furthermore, the loss of phosphorylation upon the loss of a conserved aspartate residue supported this residue as the Psyr_2449 phosphorylation site (Fig. S1).

10.1128/mBio.01178-17.1 FIG S1  Smp is phosphorylated by BphP1, and this phosphorylation requires a conserved aspartate residue in Smp. (A) The transfer of ^32^P from BphP1 to Smp increased over time. From left to right, the lanes contain BphP1 autophosphorylated in response to red light, Smp incubated in the absence of autophosphorylated BphP1 for 15 min, and autophosphorylated BphP1 and Smp coincubated for 1, 5, 10, and 15 min. (B) The transfer of ^32^P from BphP1 to Smp over time was quantified based on counts per minute, where BphP1 and Smp were coincubated for 1, 7, and 15 min, and the resulting gel (not shown) was subjected to phosphorimaging. (C) Loss of a conserved aspartate in Smp was associated with loss of phosphorylation by BphP1. From left to right, the lane contain BphP1 autophosphorylated in response to red light, BphP1 incubated in the dark, Smp incubated in the absence of BphP1, autophosphorylated BphP1 coincubated with Smp for 5 min, and autophosphorylated BphP1 coincubated with SmpD76A for 5 min. All samples were incubated in the presence of [^32^P]ATP. Download FIG S1, TIF file, 0.2 MB. Copyright © 2017 McGrane and Beattie. 2017 McGrane and Beattie This content is distributed under the terms of the Creative Commons Attribution 4.0 International license.

**TABLE 1:** Proteins predicted to interact with BphP1

Protein	Probability of interaction	Predicted domain structure and function
Psyr_2449 (Smp)	0.596	CheY-like REC domain, putatively cotranscribed with chemotaxis genes
Psyr_0886	0.181	CheY-like REC domain and CheC domain
Psyr_4376	0.138	CheY-like REC domain and LuxR-like DNA binding domain
Psyr_0258	0.053	OmpR, osmolarity response regulator
Psyr_0489	0.017	CheY-like REC domain, ortholog of a twitching motility protein, PilH, in other pseudomonads
Psyr_5032	0.007	CheY-like REC domain and DNA binding effector domain
Psyr_3091	0.004	CheY-like REC domain and DNA binding effector domain
Psyr_0488	0.004	CheY-like REC domain, ortholog of a twitching motility protein, PilG, in other pseudomonads

**FIG 3:**
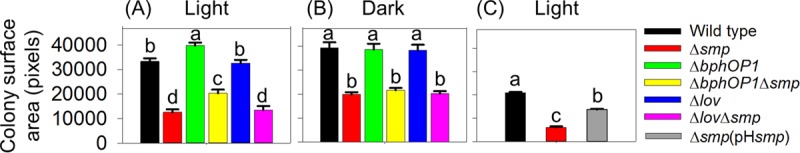
Smp, like BphP1, positively regulates swarming motility. (A to C) Swarming motility, as quantified on the basis of colony surface area, is shown for the wild-type *P. syringae* pv. syringae B728a and Δ*smp*, Δ*bphOP1*, Δ*bphOP1* Δ*smp*, Δ*lov*, and Δ*lov* Δ*smp* strains in white light (A) and dark (B) conditions and for the wild-type, Δ*smp*, and Δ*smp*(pH*smp*) strains in white light (C). Values reflect the mean colony surface areas plus SEMs (error bars) (*n* = 5), and values represented by the same lowercase letter within a panel do not differ significantly (*P* < 0.05 by two-way ANOVA with strain and replicate plate as factors). Results are representative of at least three replicate experiments.

We evaluated the swarming motility of a mutant lacking Psyr_2449. On the basis of the reduced swarming activity of this mutant, indicative of positive regulation, we designated Psyr_2449 *smp* for swarm motility-promoting regulatory gene; this gene is predicted to be part of an operon that includes several chemotaxis and RR genes. A Δ*smp* strain was significantly reduced in its ability to swarm under both light (Fig. 3A) and dark (Fig. 3B) conditions, suggesting that Smp promotes swarming motility independently of light. When *smp* was expressed under the control of a high-expression promoter on the pH*smp* plasmid in the Δ*smp* strain, swarming was at least partially restored (Fig. 3C), supporting a role for Smp in promoting swarming motility.

To determine whether the Smp regulation involves interactions with BphP1 and LOV, we evaluated the swarming motility of the Δ*bphOP1* Δ*smp* and Δ*lov* Δ*smp* double mutants. Although the Δ*smp* and Δ*smp* Δ*lov* strains were similar in their swarming in the light and dark, suggesting that Smp may act downstream of LOV, the Δ*smp* strain exhibited significantly less swarming than the Δ*bphOP1* Δ*smp* double mutant in the light (Fig. 3A), demonstrating that BphP1-mediated repression still occurs in the Δ*smp* strain. Moreover, this BphP1-mediated repression did not occur in the dark (Fig. 3B). Thus, Smp regulation occurred via a distinct pathway from BphP1 regulation, and Smp regulation did not depend on light.

**FIG 4:**
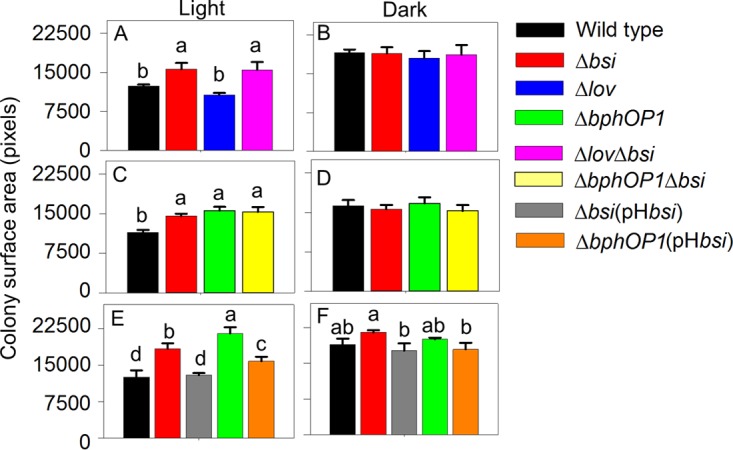
Bsi functions with BphP1 to negatively regulate swarming motility. (A to F) Swarming motility of the wild-type *P. syringae* pv. syringae B728a strain and Δ*bsi* strain, along with Δ*lov* and Δ*lov*-*bsi* strains (A and B), Δ*bphOP1* and Δ*bphOP1* Δ*bsi* strains (C and D), or Δ*bsi*(pH*bsi*), Δ*bphOP1*, and Δ*bphOP1*(pH*bsi*) strains (E and F) were evaluated under white-light (A, C, and E) and dark (B, D, and F) conditions. Values, error bars, statistical analyses, and experimental replication are as described in the legend to Fig. 3.

In another approach to identify BphP1/LOV pathway components, we investigated a gene that was predicted to be in an operon with *lov*. This gene, *Psyr_2699*, encodes a predicted integral membrane protein of unknown function. On the basis of a report of cotranscription with *lov* in *P. syringae* pv. tomato DC3000, we evaluated cotranscription in *P. syringae* pv. syringae B728a by testing for amplification of cDNA from the intergenic region between *lov* and *Psyr_2699*. We observed amplification products, but only weak ones, from the intergenic region (Fig. S2). The expression of reporter gene fusions containing the regions upstream of *lov* and *Psyr_2699* indicated that these genes have separate promoters (B. Janssen and G. A. Beattie, unpublished data), supporting the possibility that the weakly amplified intergenic region resulted from a leaky *lov* terminator. The higher swarming motility of a mutant lacking *Psyr_2699* compared to the wild type indicated that, like BphP1 (17), Psyr_2699 represses swarming motility and does so in the light, but not in the dark (Fig. 4). We designated Psyr_2699 Bsi for bacteriophytochrome-regulated swarming inhibitor. A double mutant lacking Δ*lov-bsi* (*Psyr_2699-2700*) exhibited swarming similar to that of the Δ*bsi* strain (Fig. 4A), indicating that loss of *bsi* is phenotypically dominant to loss of *lov* and that Bsi acts downstream of LOV. Although the Δ*lov* strain consistently swarmed less than the wild type, this reduction varied in significance from experiment to experiment. We speculate that this variability is due to a greater sensitivity of LOV than BphP1 to environmental conditions. Loss of both *bphP1* and *bsi* did not result in greater swarming than loss of either gene alone (Fig. 4C and D), suggesting that BphP1 and Bsi repress swarming motility through the same pathway. Overexpression of *bsi* in the Δ*bsi* and Δ*bphOP1* strains reduced swarming to wild-type levels in both strains in the light (Fig. 4E), providing further evidence that Bsi and BphP1 function in the same pathway. The wild-type, Δ*bsi*, and Δ*bphOP1* strains did not differ significantly in swarming in the dark (Fig. 4F), whereas the Δ*bsi*(pH*bsi*) and Δ*bphOP1*(pH*bsi*) strains showed reduced swarming compared to the Δ*bsi* strain; this reduction may have been due to the high level of expression of *bsi*.

10.1128/mBio.01178-17.2 FIG S2  The *lov* and *Psyr_2699* (*bsi*) genes do not appear to be cotranscribed. Cells were grown in the dark in KB medium for 5 h to a density of 4 × 10^8^ cells ml^−1^. Total RNA was extracted using the RNeasy minikit (Qiagen), contaminating DNA was removed using on-column DNase I digestion, and cDNA was synthesized using the SuperScript III First-Strand Synthesis System kit (Invitrogen, Carlsbad, CA). (A and B) PCR amplification using primer sets targeting four regions of the *Psyr_2699-2700* (*lov-bsi*) locus (see Table S1 in the supplemental material) (A) showed the presence of amplified bands in all four target regions when reverse transcriptase was present to generate cDNA, but not when reverse transcriptase was absent (B). The amplification of fragments using primer sets 2 and 3 with cDNA but not RNA indicated that the intergenic region was transcribed, but the lower-intensity bands from the intergenic region compared to the those from the intragenic regions support weak transcriptional termination from *lov* rather than a polycistronic transcript, or alternatively, transcription as a polycistronic mRNA followed by posttranscriptional modification or secondary structure that reduces the efficiency of PCR amplification. Download FIG S2, TIF file, 0.1 MB. Copyright © 2017 McGrane and Beattie. 2017 McGrane and Beattie This content is distributed under the terms of the Creative Commons Attribution 4.0 International license.

**FIG 5:**
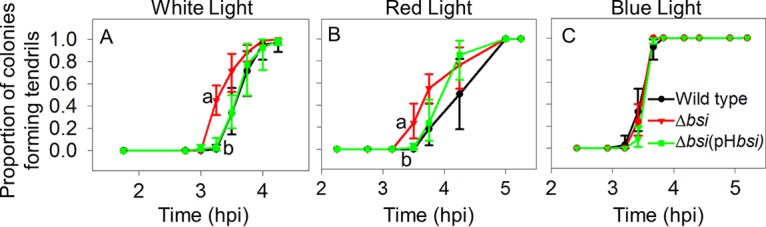
Bsi regulates swarming initiation in response to red, but not blue, light. The timing of tendril formation was compared in the wild-type *P. syringae* pv. syringae B728a, Δ*bsi*, and Δ*bsi*(pH*bsi*) strains in white light (A), red light (B), and blue light (C). Values, error bars, statistical analyses, experimental replication, and abbreviations are as described in the legend to Fig. 1.

We also evaluated the timing of tendril initiation in Δ*bsi*, Δ*bsi*(pH*bsi*), and wild-type strains. In both white and red light, the Δ*bsi* strain initiated tendril formation earlier than the wild type did, while the Δ*bsi*(pH*bsi*) strain initiated tendril formation similar to the wild type (Fig. 5A and B). In contrast, in blue light, the Δ*bsi* and Δ*bsi*(pH*bsi*) strains initiated tendril formation similar to the wild type (Fig. 5C). Bsi, like BphP1, therefore regulates tendril initiation in response to red, but not blue, light.

### A quorum molecule that negatively regulates tendril initiation functions downstream of BphP1 and Bsi.

**FIG 6:**
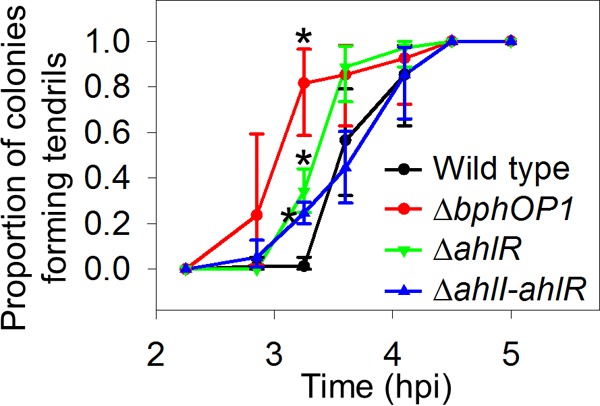
The contribution of acyl-homoserine lactone to regulation of swarming initiation is smaller than that of BphP1. Swarm tendril initiation was measured as described in the legend to Fig. 1. Values with an asterisk are those in which the mutant differs significantly from the wild-type *P. syringae* pv. syringae B728a at that time point (*P* < 0.05 by unpaired Student’s *t* test on arcsine-transformed data in comparisons between a single mutant and the wild type).

**FIG 7:**
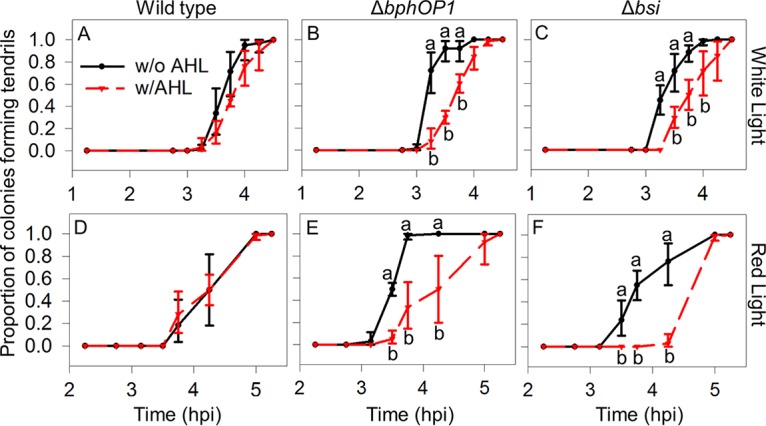
Addition of an acyl-homoserine lactone (AHL) reduces the time to swarming initiation for the Δ*bphOP1* and Δ*bsi* strains, but not the wild type, in white and red light. (A to F) The time until swarming was initiated was examined for the wild-type *P. syringae* pv. syringae B728a (A and D), Δ*bphOP1* strain (B and E), and Δ*bsi* strain (C and F) with 0.1 μM *N*-(β-ketocaproyl)-l-homoserine lactone (w/AHL) and without AHL (w/o AHL) under white light (A to C) and red light (D to F). Values indicated by the same lowercase letter do not differ significantly for comparisons within a single time point (*P* < 0.05 by two-way ANOVA on arcsine-transformed data where strain and the presence of AHL were factors). Values, error bars, experimental replication, and abbreviations are as described in the legend to Fig. 1.

The *N*-acyl-homoserine lactone (AHL) quorum molecule produced by *P. syringae* pv. syringae B728a, 3-oxo-hexanoyl homoserine lactone, is involved in swarming initiation and formation of water-soaked lesions (32). Mutants lacking either the regulator AhlR or both AhlR and the AHL synthase AhlI initiated tendril formation earlier than the wild type did (Fig. 6), much like a mutant lacking the AHL regulator AefR in a previous study (32). However, these mutants did not initiate swarming as early as the Δ*bphOP1* strain did, suggesting that if AHL regulation occurs in the BphP1 pathway, then it occurs downstream of BphP1. Amendment of the inoculum with a commercial AHL, *N*-(β-ketocaproyl)-l-homoserine lactone, did not significantly alter the behavior of the wild type but delayed swarming initiation by the Δ*bphOP1* and Δ*bsi* strains in both white and red light (Fig. 7). The response of the Δ*bphOP1* and Δ*bsi* strains to the addition of the AHL (Fig. 7) coupled with the behavior of the Δ*ahlI-ahlR* and Δ*ahlR* strains (Fig. 6) support a model in which BphP1 and Bsi repress swarming initiation by regulating AHL production.

### BphP1 and Bsi, but not Smp, negatively regulate virulence on beans.

**FIG 8:**
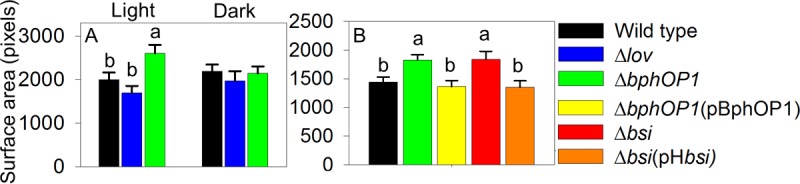
BphP1 and Bsi negatively regulate virulence, as indicated by lesion induction on bean pods, in a light-dependent manner. Lesion sizes were quantified in digitized images taken 36 to 48 h following wound inoculation of bean (*Phaseolus vulgaris*) pods. (A and B) Lesion sizes were compared for the wild type and Δ*bphOP1* strain, along with the Δ*lov* mutant in white-light and dark conditions (A), or Δ*bphOP1*(pBphOP1), Δ*bsi*, and Δ*bsi*(pHbsi) strains in light conditions (B). Values represent the mean lesion areas plus SEMs (*n* = 9 for panel A; *n* = 8 for panel B). Values that do not differ significantly are indicated by the same lowercase letter within a panel (*P* < 0.05 by two-way ANOVA with strain and replicate experiments as factors in panel A; *P* < 0.05 by one-way ANOVA in panel B). Results are combined means of three replicate experiments or representative of three replicate experiments for panels A and B, respectively.

To investigate whether BphP1 and LOV contribute to lesion formation, we inoculated bean (*Phaseolus vulgaris*) pods with the wild-type, Δ*lov*, Δ*bphOP1*, and Δ*bphOP1*(pBphOP1) strains by injection and incubated the pods in either light or dark conditions for 36 to 48 h. The Δ*bphOP1* strain induced water-soaked lesions that were significantly larger than those of the wild type in the light (Fig. 8A), with this regulation specific to light conditions (Fig. 8A) and the loss of *bphOP1* complemented by overexpression of *bphOP1* (Fig. 8B). In contrast, the Δ*lov* strain formed lesions that were consistently smaller in our experiments, although these reductions were not significant (Fig. 8A). This BphP1- and LOV-mediated regulation of virulence on bean pods resembles BphP1- and LOV-mediated regulation of swarming motility.

To determine whether Bsi or Smp act together with BphP1 to regulate virulence, we injected the wild-type, Δ*bsi*, Δ*bsi*(pH*bsi*), Δ*smp*, Δ*smp*(pH*smp*), Δ*bphOP1*, and Δ*bphOP1*(pBphOP1) strains into bean pods and incubated them in the light. Like the Δ*bphOP1* strain, the Δ*bsi* strain formed larger water-soaked lesions than the wild type did (Fig. 8B), and introducing the pH*bsi* plasmid into the Δ*bsi* strain reduced lesion formation to wild-type levels. In contrast, the Δ*smp* and Δ*smp*(pH*smp*) strains induced lesions that did not differ from those induced by the wild type (Fig. S3), demonstrating that Bsi, but not Smp, is involved in regulating virulence.

10.1128/mBio.01178-17.3 FIG S3  Smp does not influence lesion development on bean pods. (A) Lesion sizes of the wild-type, Δ*bphOP1*, Δ*bphOP1*(pBphOP1), Δ*smp*, and Δ*smp*(pH*smp*) strains were compared at 36 to 48 h after inoculation and incubation in white light. Values and error bars (*n* = 9) are as described in the legend to Fig. 3 (*P* < 0.05 by one-way ANOVA). Download FIG S3, TIF file, 0.04 MB. Copyright © 2017 McGrane and Beattie. 2017 McGrane and Beattie This content is distributed under the terms of the Creative Commons Attribution 4.0 International license.

### BphP1 negatively regulates movement of *P. syringae* to seeds in soil.

**FIG 9:**
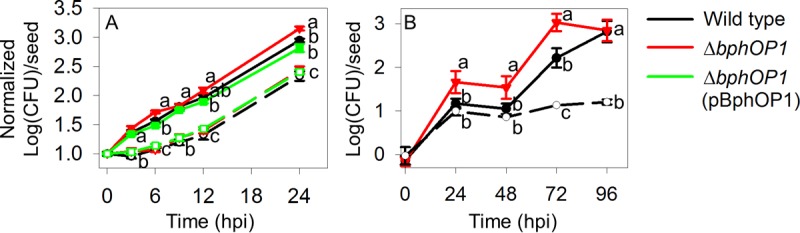
BphP1 negatively regulates movement in soil from bulk soil or infected leaf tissue to seeds. Bacterial populations were measured on seeds after planting in soils that contained target *P. syringae* strains and on seeds after the seeds had been placed in soils with a target *P. syringae* strain for a short period and then transferred to soils without *P. syringae*; the latter provided a mechanism for evaluating seed-borne bacterial growth in the absence of bacterial movement to the seed. (A) Populations of the wild-type *P. syringae* pv. syringae B728a, Δ*bphOP1*, and Δ*bphOP1*(pBphOP1) strains were monitored for 24 h on seeds planted in sand inoculated with suspensions of each strain and on seeds after a 7-min incubation in inoculated sand and subsequent transfer to uninoculated, sterilized sand. (B) The populations of the wild type and Δ*bphOP1* strain were monitored for 96 h on seeds planted in the field following soil amendment with infected leaf tissues and on seeds after a 1-h incubation in soil amended with *P. syringae* pv. syringae B278a-infected leaf tissues and subsequent transfer to unamended field soil. For both the laboratory (A) and field (B) tests, “transferred” refers to the populations on the seeds that had been transferred to uninoculated/unamended soils. Samples that remained in inoculated soil for the duration of the experiment are shown as solid lines with filled symbols, whereas transferred samples are shown as broken lines with open symbols. The log(CFU) seed^−1^ values were normalized based on the mean populations on the seeds at 0 hpi, and the values shown are the mean normalized log(CFU) seed^−1^ and SEM values (*n* = 8 for panel A; *n* = 16 for panel B). Comparisons were made within each time point (*P* < 0.05 by one-way ANOVA). Results are representative of two replicate experiments for both panels (A and B).

An important step in the life cycle of *P. syringae* pv. syringae B728a is transmission from infected leaves, which have overwintered in the soil, to seeds and seedlings in the spring; this transmission may involve active movement and thus swarming motility. To test this, we inoculated sterilized sand with cultures of the wild-type, Δ*bphOP1*, and Δ*bphOP1*(pBphOP1) strains. After we planted bean seeds in the inoculated sands, we monitored the populations on the seeds for 24 h. To assess bacterial growth in the absence of movement to the seeds, we incubated one set of seeds for 7 min in the inoculated sand and then transferred the seeds to uninoculated sterilized sand, thus allowing us to monitor growth of the inoculum that was initially acquired by the seed in the absence of subsequent bacterial movement to the seeds. We found that the populations on seeds in inoculated soil were significantly higher than on seeds that were transferred to uninoculated soil (Fig. 9A). This finding provides evidence of *P. syringae* movement in soil to seeds. The populations of the three strains were similar on seeds in the uninoculated sand (Fig. 9A), indicating that the different strains grew at comparable rates on the seeds. The populations of the Δ*bphOP1* strain on seeds in the inoculated sand, however, were larger than those of both the wild-type and Δ*bphOP1*(pBphOP1) strains at 24 h after planting (Fig. 9A), illustrating a role for BphP1 in repressing movement in soil.

We also performed this experiment under field conditions over a 4-day period using inocula comprised of dried tissue samples from bean leaves that had been infected with the wild-type or Δ*bphOP1* strain; dried leaves were added to the soil to mimic natural inoculum. To evaluate bacterial growth in the absence of movement, we incubated one set of seeds for 1 h in field soil amended with dried leaf tissues infected with the wild-type strain and then transferred the seeds to unamended field soils. The wild-type populations on the seeds in amended soils were significantly higher than those on seeds following transfer to the unamended soils (Fig. 9B), again providing evidence of *P. syringae* movement in soil, but this time from infected leaf tissues to seeds. Moreover, the Δ*bphOP1* strain established significantly higher populations by 24, 48, and 72 h than the wild type did (Fig. 9B), demonstrating a role for BphP1 and supporting a role for swarming motility in *P. syringae* movement in soil.

### BphP1, but not Bsi or Smp, contributes to leaf colonization.

**FIG 10:**
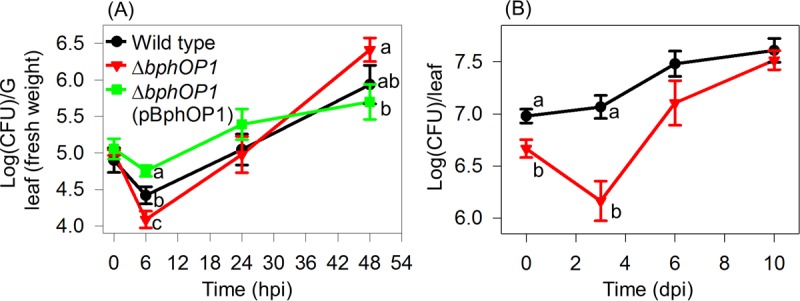
BphP1 positively contributes to survival in early stages of colonization (<10 h) and negatively regulates colonization in later stages (>24 h). Bacteria were inoculated onto bean leaves, and leaf-associated populations were monitored via recovery by leaf homogenization and plating on selective media. (A) Plants were inoculated with the wild-type *P. syringae* pv. syringae B728a, Δ*bphOP1*, and Δ*bphOP1*(pBphOP1) strains and incubated in a growth chamber. Values are mean log(CFU) g^−1^ ± SEM values (*n* = 5 to 12), with comparisons made within a time point (*P* < 0.05 at 6 h and *P* < 0.1 at 48 h by one-way ANOVA). (B) Plants were inoculated with the wild type and Δ*bphOP1* strain on bean plants in the field. Values are mean log(CFU) g^−1^ ± SEM values (*n* = 16), with comparisons made within a time point (*P* < 0.05 in a Student’s *t* test). dpi, days postinoculation. Results are representative of at least three replicate experiments.

We also examined the impact of BphP1 on leaf colonization by monitoring populations on bean leaves following inoculation by leaf submersion and incubation with a day/night cycle using white lights in a growth chamber. Loss of *bphOP1* reduced populations at 6 hpi (Fig. 10A), whereas overexpression of *bphOP1* increased populations by 6 hpi relative to the wild type and reduced populations by 48 hpi relative to the Δ*bphOP1* strain (Fig. 10A). When the data from three replicate experiments were collectively analyzed using a repeated measures analysis, the population of the Δ*bphOP1* strain was significantly higher than those of the wild type (*P* = 0.03) and Δ*bphOP1*(pBphOP1) strain (*P* = 0.0005) at 48 h. The Δ*bphOP1* strain also exhibited an initial reduction in population size following inoculation and subsequent growth to wild-type levels in studies conducted in the field (Fig. 10B). These findings indicate that BphP1 contributes to survival in the hours following inoculation onto leaves but negatively affects leaf colonization at later stages. Following inoculation onto leaves, populations of the Δ*bsi*, Δ*bsi*(pH*bsi*), Δ*smp*, and Δ*smp*(pH*smp*) strains were similar to those of the wild type over a 4-day time course (Fig. S4), demonstrating that neither Bsi nor Smp contributes to B728a leaf colonization.

10.1128/mBio.01178-17.4 FIG S4  Smp and Bsi do not contribute to leaf colonization. Total population sizes of the wild-type *P. syringae* pv. syringae B728a, Δ*smp*, Δ*smp*(pH*smp*), Δ*bsi*, and Δ*bsi*(pH*bsi*) strains were evaluated at 6, 24, and 48 h after inoculation onto leaves. Bacteria were recovered by leaf homogenization. Values and error bars (*n* = 12) are as described in the legend to Fig. 10 (*P* < 0.05 by one-way ANOVA). Download FIG S4, TIF file, 0.1 MB. Copyright © 2017 McGrane and Beattie. 2017 McGrane and Beattie This content is distributed under the terms of the Creative Commons Attribution 4.0 International license.

## DISCUSSION

In this work, we provide evidence for a role of the red/far-red-sensing photosensory protein BphP1 in multiple stages of the life cycle of the foliar pathogen *P. syringae* pv. syringae B728a, thus illustrating that red/far-red light perception influences the biology of this pathogen. In particular, we found that the bacteriophytochrome BphP1 negatively regulates virulence, as reflected in its ability to restrict the size of water-soaked lesions on bean pods, and negatively regulates movement through soil to seeds. Interestingly, while BphP1 negatively regulates traits contributing to the establishment of large populations on leaves, it positively regulates traits that contribute to survival in the hours following leaf inoculation. One or more of these plant and soil phenotypes may be phenotypically linked to BphP1-mediated repression of swarming. We identified two components of the BphP1-mediated signal transduction pathway: a protein encoded by a gene that is adjacent to *lov*, which we designated Bsi, and the AHL quorum molecule synthesized by the B728a strain. Last, we elucidated at least one mechanism by which BphP1 represses swarming motility, and that is by repressing the initiation of the tendrils that extend outward from a swarm colony. The role of the BphP1-Bsi-AHL pathway in delaying tendril initiation, which is critical to swarming motility by strain B728a, suggests that this pathway represses a switch from a sessile lifestyle to a motile lifestyle.

**FIG 11:**
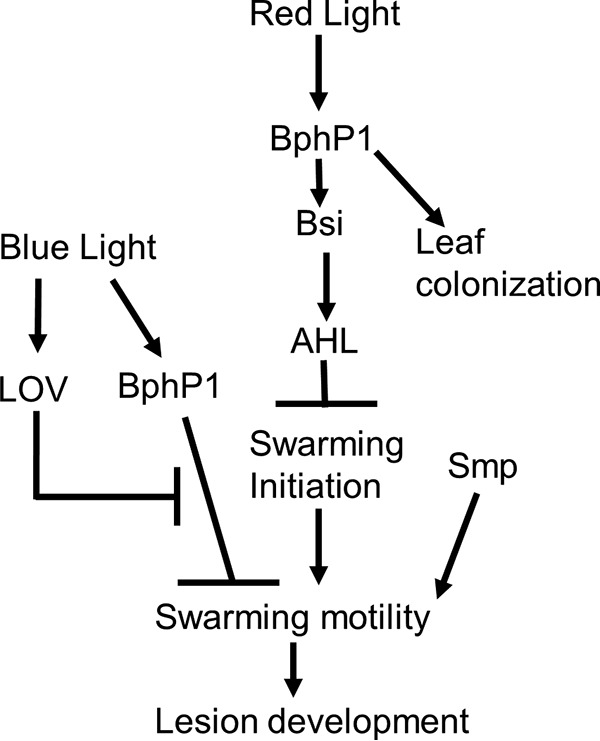
Model of the swarming regulation by BphP1, Bsi, and Smp. BphP1 and Bsi repress swarming motility in response to red light, in part through their regulation of AHL production, although they may also regulate swarming motility through other mechanisms. Smp regulates swarming motility through an independent pathway in response to an unknown stimulus.

Our results support a model in which BphP1 represses swarming motility by delaying swarming initiation in response to red light and does so, at least in part, by acting through Bsi to activate AHL synthesis (Fig. 11). Multiple lines of evidence support this branch of the model, including the following. (i) Mutants lacking *bsi*, like those lacking *bphP1*, exhibited hyperswarming in a light-dependent manner. (ii) *bphP1* and *bsi* mutants initiated swarming earlier than the wild type did in a red-light-dependent manner. (iii) The *ahlI-ahlR* and *ahlR* mutants, which lack a positive regulator of AHL synthesis, initiated swarming earlier than the wild type did but not as early as the *bphP1* mutant did. (iv) AHL amendment delayed swarming initiation by mutants lacking *bphOP1* or *bsi* in a red-light-dependent manner. The lack of an effect of AHL amendment on swarming initiation by the wild type is likely because its endogenous level of AHL is maximal due to BphP1/Bsi-mediated AHL production coupled with autoregulation of AHL production (37). These results are consistent with previous observations correlating loss of AHL production with hyperswarming and early initiation of swarming in *P. syringae* and *P. aeruginosa* (32, 38). Regulation of swarming motility by BphP1 and Bsi is probably not due solely to regulation of tendril initiation, because repression of tendril initiation by the addition of AHL did not completely abolish hyperswarming in mutants lacking *bsi* and *bphP1* (data not shown).

The BphP1-Bsi-AHL pathway negatively regulates the expansion of water-soaked lesions on bean pods, as demonstrated by the increased size of the lesions generated by mutants lacking *bphP1* or *bsi*, and also *ahlR* and *ahlI*-*ahlR* as observed in a previous study (32). The simplest explanation for a role for BphP1, Bsi, and AHL in lesion formation is via regulation of swarming motility; increased swarming motility could enable access to a more extensive region of plant tissue, thus resulting in larger water-soaked lesions. Alternative explanations include an influence on other traits contributing to water soaking. For example, the induction of water-soaked lesions in the leaf mesophyll was recently associated with production of the effector proteins AvrE and HopM1 in *P. syringae* pv. tomato DC3000 (39). These proteins, which are present in *P. syringae* pv. syringae B728a, could also contribute to water soaking in bean pods, although this has not been tested. Alternatively, the BphP1-Bsi-AHL pathway could influence the production of enzymes involved in tissue maceration based on a correlation between loss of *ahlR* and *ahlI-ahlR* and reduced tissue maceration in bean pods (32).

BphP1 negatively regulates *P. syringae* movement in soil, and although we did not test the light dependency of this regulation, the involvement of BphP1 supports red- or far-red-light penetration of a soil matrix. Light penetration of soil matrices is known (40), with greater penetration exhibited by long wavelengths, especially far-red light (41). The biological relevance of this penetration includes an impact on seed germination (42). Moreover, once a seed germinates, the piping of far-red light through the stem and the roots (43, 44) could provide an even greater abundance of light as a belowground signal in the root zone. Interestingly, strain B728a BphP1 negatively regulated movement from the soil to seeds, indicating that far-red light is not serving as an activator of motility for attraction to the seed or seedling. Alternatively, we speculate that far-red light may function as a cosignal for conditions of low water availability, since low water availability enables greater light scattering in soil matrices (40, 41), and this would effectively increase the local availability of light; such cosignaling would reinforce that the low-water conditions are unfavorable for movement.

We found that the contribution of BphP1 to leaf colonization is complex. Following inoculation onto leaves under dry conditions, *P. syringae* pv. syringae B728a populations decrease, in part due to desiccation stress; loss of *bphP1* decreased survival during this period. This reduction in survival could be due to hyperswarming or reduced AHL production by mutants lacking *bphP1*, both of which could reduce the formation of protective aggregates. Previous studies with strain B728a have associated AHL production with alginate synthesis, the formation of cellular aggregates, and aggregation-mediated tolerance to desiccation stress on leaves (32, 37, 45). In the period following inoculation onto leaves, when B728a cells have not yet entered into internal leaf sites, and thus B728a cells must survive primarily on the leaf surface, hyperswarming and a deficiency in alginate production due to loss of BphP1-mediated AHL production would likely reduce bacterial tolerance to the stresses associated with drying, and thus reduce bacterial survival. This reduced survival was reported for an *ahlI-ahlR* mutant of strain B728a (32). The light-mediated repression of swarming motility in strain B728a may enable it to remain motile on leaves in the dark, which is a period when leaf surfaces are generally moist. In contrast to decreased survival in the hours after inoculation, the loss of *bphP1* was associated with eventual increases in populations on leaves; this growth was likely in epiphytic and internal leaf sites. Increased populations could be due to hyperswarming enabling greater access to nutrients, since nutrients are relatively localized on leaf surfaces (46). Hyperswarming could additionally enhance access to internal leaf sites. Importantly, Bsi and Smp both contribute to regulation of swarming motility, but neither affected early or late-stage leaf colonization (see Fig. S3 in the supplemental material). This provides strong evidence that some BphP1-mediated contributions to leaf colonization are, at least in part, independent of swarming motility and of Bsi (Fig. 11).

Smp is an additional regulator of swarming motility that interacts with BphP1 *in vitro*, but it does not regulate swarming motility via the same pathway as BphP1 does and does not influence the other BphP1-mediated phenotypes examined. A bioinformatic search and a screen for phosphorylation targets of BphP1 identified Smp as a potential component of the BphP1-mediated signal transduction pathway, but the swarming motility of a *smp* mutant and *smp bphOP1* double mutant illustrated that BphP1 and Smp regulate swarming through independent pathways. Moreover, Smp was not involved in BphP1-mediated regulation of lesions or leaf colonization. Given the ability of BphP1 to phosphorylate Smp, BphP1-mediated attenuation of Smp-activated swarming is possible, as it would likely have been obscured by Smp-independent BphP1 repression of swarming in these assays. However, the *in vitro* interaction of Smp and BphP1 could also be due to nonspecific cross talk, as histidine kinases have a strong propensity to phosphorylate alternative targets in the absence of their cognate response regulators (47).

Collectively, this work helps elucidate the physiological role of BphP1 in *P. syringae* pv. syringae B728a and provides evidence for bacteriophytochrome-mediated regulation of *P. syringae* phenotypes affecting virulence and plant colonization. The involvement of Bsi in some but not all of the BphP1-mediated phenotypes demonstrates branching of the BphP1-regulated pathway. While Bsi, a novel protein involved in swarming motility, acts downstream of BphP1 in regulating swarming, Bsi is not the cognate response regulator for BphP1, as it does not have a response regulator domain that could serve as a phosphoryl receiver. Thus far, the identities of bacteriophytochrome-interacting response regulators have remained elusive; however, the identification of additional bacteriophytochrome pathway components, as was done here, brings us one step closer to elucidating the full red/far-red-light-responsive pathway. This information is important to understanding the molecular mechanisms by which light signals are transduced into phenotypes relevant to the life cycles and virulence traits of plant pathogens and the extent to which bacteriophytochromes have evolved similar signal transduction pathways in distinct bacteria.

## MATERIALS AND METHODS

### Bacterial strains and growth conditions.

The bacterial strains and plasmids for this study are described in Table 2. *P. syringae* strains were grown in King’s B (KB) medium (48) at 25°C unless otherwise described. *Escherichia coli* strains were grown in Luria medium at 37°C. The following antibiotics were added at the indicated concentrations as needed: rifampin (Rif), 50 µg ml^−1^; kanamycin (Km), 50 µg ml^−1^; chloramphenicol (Cm), 30 µg ml^−1^; cycloheximide (Cyclo), 100 µg ml^−1^; and ampicillin (Amp), 50 µg ml^−1^.

**TABLE 2:** Bacterial strains and plasmids used in this study

Strain^a^ or plasmid	Description and/or relevant genotype or phenotype	Reference or source
Strains		
B728a	*P. syringae* pv. syringae; wild type; Rif^r^	50
Δ*bphOP1*	B728a Δ*Psyr_3505-3504*; Rif^r^	17
Δ*lov*	B728a Δ*Psyr_2700*; Rif^r^	17
Δ*smp*^b^	B728a Δ*Psyr_2449*; Rif^r^	This study
Δ*bsi*^b^	B728a Δ*Psyr_2699*; Rif^r^	This study
Δ*lov-bsi*	B728a Δ*Psyr_2700-2699*; Rif^r^	This study
Δ*ahlR*	B728a Δ*Psyr_1622*::*km*; Rif^r^ Km^r^	37
Δ*ahlI-ahlR*	B728a Δ*Psyr_1621-1622*::*km*; Rif^r^ Km^r^	37

Plasmids		
pME6041	Broad-host-range vector; Km^r^	51
pN	pME6041 with *nptII* promoter next to the multiple cloning site; Km^r^	52
pBphOP1	pN with *bphOP1* under the control of the *nptII* promoter in tandem with the *bphO* promoter; Km^r^	17
pH	pME6041 with the high-expressing *Psyr_1321* promoter; Km^r^	This study
pH*smp*	pH with *Psyr_2449*; Km^r^	This study
pH*bsi*	pH with *Psyr_2699*; Km^r^	This study
pET21a^c^	Vector for inducible expression of C-terminal His_6_-tagged proteins; Ap^r^	EMD Biosciences
pET21a-*bphP1*	pET21a with Psyr_3504; Ap^r^	This study
pET21a-*smp*	pET21a with Psyr_2449; Ap^r^	This study
pET21a-*smp*D76A	pET21a with a point mutation changing Asp to Ala at amino acid 76; Ap^r^	This study

^a^ Bacterial strains are indicated by their strain designation or relevant genotype.

^b^ The Δ*bsi* and Δ*smp* strains were used for constructing the Δ*bphOP1* Δ*smp*, Δ*lov* Δ*smp*, and Δ*bphOP1* Δ*bsi* double mutants.

^c^ The following genes were also cloned into pET21a: *Psyr_0488*, *Psyr_0489*, *Psyr_0886*, *Psyr_3299*, *Psyr_3433*, *Psyr_4376*, and *Psyr_4392*.

### Construction of mutants and plasmids for complementation.

Deletion mutants were generated by splice-overlap-extension PCR mutagenesis as previously described (17) and using the primers shown in Table S1 in the supplemental material. Overexpression and complementation plasmids were constructed by cloning PCR amplified fragments of *bsi* and *smp* into the SmaI site of the pH vector, which was pME6041 with a 246-bp promoter region of *Psyr_1321* inserted upstream of the multiple cloning site. *Psyr_1321* was identified by microarray analysis to be a highly expressed, constitutive promoter expressing a gene for a hypothetical protein; in particular, *Psyr_1321* was expressed at a level higher than 85% of the *P. syringae* pv. syringae B728a genes in a basal medium and exhibited little change in expression in response to growth *in planta* or in a variety of stressful conditions (49).

10.1128/mBio.01178-17.5 TABLE S1  Primers used for this study. Download TABLE S1, DOCX file, 0.01 MB. Copyright © 2017 McGrane and Beattie. 2017 McGrane and Beattie This content is distributed under the terms of the Creative Commons Attribution 4.0 International license.

### Assay for quantifying swarming motility.

Analysis of swarming motility was performed as previously described (17). Cells grown in KB to late-log phase were washed, and a 2-µl suspension of 8 × 10^5^ cells was inoculated onto swarm plates (KB with 0.4% agar) using the plate design described by Wu et al. (17). Briefly, swarm plates were inoculated with five replicate cultures of up to five strains, and the plates were either enclosed in two layers of aluminum foil to create dark conditions or were exposed to 30 µmol m^−2^ s^−1^ of continuous white light. The plates were incubated side by side at 22-23°C for 10 to 14 h and were photographed when swarm colonies were still at least 5 mm apart. The surface area of each colony was quantified and analyzed as described previously (17).

### Assay for quantifying swarming initiation.

The initiation of swarming was designated as the time at which swarm tendrils were first detected as bulges along the circular colony edge during growth on swarm plates. Swarm plates and inocula were prepared as described above, except that 4 × 10^6^ cells were inoculated onto each plate. The plates were exposed to dark conditions and white light as described above, to 21 µmol m^−2^ s^−1^ of blue light (470 nm) using bilirubin bulbs (Bili Blue; Interlectric Corp., Warren, PA), and to 6.5 µmol m^−2^ s^−1^ of red/far-red light (680 and 750 nm) using F40T12R bulbs (Interlectric Corp., Warren, PA). For each colony, visible tendril initiation was recorded at 15- to 30-min intervals starting 2 to 3 h after inoculation; scoring was performed blind to the strain identity of each colony. At each time point, the proportion of colonies of a strain that scored positive on each of four plates was calculated. The arcsine-transformed values were used to estimate the mean, the standard error of the mean (SEM), and statistical significance, with the back-transformed values represented in the figures. To determine the role of an AHL signal molecule in swarming initiation, purified *N*-(β-ketocaproyl)-l-homoserine lactone (Sigma-Aldrich Corp. St. Louis, MO) was added to the inoculum cells to a final concentration of 0.1 µM immediately before placing the cells on the plates.

### Identification and analysis of candidate BphP1-interacting proteins.

Response regulators that may interact with BphP1 were selected using Prediction of Interaction Specificity of Two-component Systems software (Swiss Institute of Bioinformatics) (35). Tagged derivatives of BphP1 and eight of these putative BphP1-interacting proteins, Psyr_2449 (Smp), Psyr_0886, Psyr_4376, Psyr_0489, Psyr_0488, Psyr_3433, Psyr_4392, and Psyr_3299, were generated by inserting the genes into the multiple cloning site of pET21a to create C-terminal His_6_-tagged fusions (Table 1). Site-directed mutagenesis of pET21-*smp* was performed using a QuikChange II XL site-directed mutagenesis kit (Agilent Technologies, Santa Clara, CA), creating pET21-*smp*D76A. The constructs were introduced independently into the protein expression strain BL21(DE3) Codon-plus-RILP (Agilent Technologies), cells were grown to late-log phase with 10 mM isopropyl-β-d-thiogalactopyranoside (IPTG) at 20°C for 16 h. His_6_-tagged proteins were purified by centrifugation at 5,000 × *g* for 10 min at 4°C, and the pellet was suspended in 20 ml of extraction buffer (300 mM NaCl, 50 mM NaH_2_PO_4_, and 10 mM imidazole [pH 8] with the addition of 200 µl phenylmethylsulfonyl fluoride [PMSF], 200 µl Triton X-100, 14 µl β-mercaptoethanol, and 200 µl protease inhibitor cocktail). Lysis was performed by exposing the cells to 12 cycles of sonication for 10 s and incubating on ice for 30 s. Cells were centrifuged again, and the lysate was applied to a PerfectPro nickel-nitrilotriacetic acid (Ni-NTA) agarose nickel affinity column (5 Prime Inc., Gaithersburg, MD) in the dark at 4°C. The column was washed three times with washing buffer (300 mM NaCl, 50 mM NaH_2_PO_4_, 20 mM imidazole [pH 8]), and the protein was eluted with elution buffer (300 mM NaCl, 50 mM NaH_2_PO_4_, 250 mM imidazole [pH 8]).

To evaluate BphP1 phosphorylation of the candidate response regulators, autophosphorylated BphP1 was prepared by exposing 10 µl of purified BphP1 (12 µM) to 10 µmol m^−2^ s^−1^ of red light (680 nm) using light-emitting diodes (Marubeni America Corporation, New York, NY) in the presence of a 10-fold molar excess of biliverdin (Frontier Scientific, Logan, UT). After 10 min, 10 µl of reaction buffer was added that contained 50 mM Tris-HCl (pH 7.6), 4 mM MgCl_2_, 100 mM KCl, 0.4 mM EDTA, 2 mM dithiothreitol, 0.04 mM ATP, and 0.2 µM [γ-^32^P]ATP. The samples were incubated for 5 min, and an equimolar concentration of a purified candidate response regulator was added to the samples. The samples were incubated for 5 min, and 20 µl of sample buffer containing 62.5 mM Tris-HCl (pH 6.8), 25% glycerol, 2% SDS, 0.01% bromophenol blue, and 5% β-mercaptoethanol was added to each sample. The protein mixtures were subjected to SDS-PAGE using Novex 12% Tris-glycine Midi gels (Life Technologies, Grand Island, NY) and fixed by washing in 40% methanol and 7% acetic acid for 15 min. The gel was subjected to phosphorimaging (PharosFX Plus Molecular Imager; Bio-Rad, Hercules, CA) to detect radiolabeled proteins and to stain with Coomassie blue to visualize all of the proteins. Radioactivity in selected gel bands was measured in counts per minute using a liquid scintillation counter (Tri-carb 2100 TR; Packard BioScience Company, Meriden, CT).

### Assay for quantifying lesion development on bean pods.

Bean (*Phaseolus vulgaris*) pods from a grocery store were sterilized in 2% sodium hypochlorite for 10 min, rinsed in sterile water, and placed on moistened filter paper in a glass tray. Late-log-phase cells were washed, and a 2-µl suspension of 8 × 10^5^ cells was inoculated into the bean pods by placing the cell suspension into a 2-mm-deep hole formed by a pipette tip puncture. The glass tray was covered with plastic wrap, enclosed in aluminum foil to create dark conditions or exposed to white light, and incubated at 22-23°C for 36 to 48 h. The surface area of each water-soaked lesion was quantified based on pixel counts in a digitized image, as described for swarming. Within an experiment, each bean pod was inoculated with each of the strains to be compared, and sufficient bean pods were used to examine at least two replicate bean pods for each of three independent cultures of each strain in each experiment.

### Quantification of bacterial movement from bulk soil or infected leaf tissues to seeds.

Late-log-phase cells were washed, and a 5-μl suspension of 8 × 10^5^ cells was placed at the center of each of three swarm plates. The plates were incubated in the dark at 22 or 23°C until the cells had swarmed over most of the plate (~16 h). The cells from the three plates were suspended in water and mixed with washed, sterilized sand (Premium Play Sand; Quikrete Cement & Concrete Products, Columbus, OH) to a final concentration of 5 × 10^2^ cells g^−1^ of sand in aluminum trays (21.5 by 28 by 5 cm). Bean seeds (*P. vulgaris* cultivar Bush Blue Lake 274 [cv. BBL274]) were placed in the sand at a depth of 1 cm. To estimate growth on seeds in the absence of movement, a subset of the seeds was left in the inoculated sand for 7 min and then transferred to uninoculated sterilized sand. The trays were placed under white lights at 20°C for 24 h, during which eight seeds per strain were collected at each sampling time, and the population on each seed was estimated by sonicating for 7 min in washing buffer (10 mM phosphate buffer [pH 7] and 5 g liter^−1^ proteose peptone) and enumerating the recovered cells on KB medium containing Rif and Cyclo. The populations were expressed as CFU seed^−1^ and were log transformed before analysis, with the samples at time zero that were below the detection limit estimated as 0.5 CFU seed^−1^.

Bacterial movement from infected leaf tissues to bean seeds in the soil was evaluated under field conditions. Infected leaf tissues were generated by inoculating 2-week-old bean plants (cv. BBL274) by leaf immersion in inoculum containing late-log-phase, KB-grown cells suspended to a density of 4 × 10^6^ cells ml^−1^ in 10 mM phosphate buffer (pH 7) (PB). The plants were incubated at room temperature with a 12-h photoperiod until disease symptoms developed, at which time the leaves were collected and cut into squares (~1 by 1 mm^2^) and the bacterial density (CFU g^−1^ of fresh leaf tissue) was measured by plating. Soil was collected from the field site at the Iowa State University (ISU) Horticulture Research Farm near Gilbert, IA, and for each bacterial strain tested, 6.5 kg of soil was amended with the infected leaf tissue to a final density of 1 × 10^5^ cells g^−1^ of soil. After the soil and infected leaf tissue were mixed, the soil was covered and incubated at room temperature overnight, then five 1-g samples were collected to evaluate the bacterial density and homogeneity of the distribution of infected plant tissue. In the field, inoculated soil was introduced into holes (2.5 cm wide, 5 cm deep), and one seed (cv. BBL274) was planted at a depth of 1 cm in each hole and provided 5 ml of sterile water. A randomized block design was used in which 22 seeds for each strain were present within each of four blocks, with the seeds spaced approximately 5 cm apart and associated with a marker stake to aid in recovery. To estimate *P. syringae* growth on seeds in the absence of movement, a treatment was included in which seeds were left in the inoculated soil for 1 h and then transferred to holes containing soil that had not been amended with infected leaf tissue. For each strain at each time point, 4 seeds were collected from each block, for a total of 16 seeds per strain. Each seed was sonicated for 7 min in washing buffer, and populations were enumerated on KB medium containing Rif and Cyclo.

### Bacterial enumeration on leaves.

Inocula containing cells from swarm plates were prepared as described above for the quantification of bacterial movement from soil to seeds, except that the cells from three swarm plates were suspended to a density of 4 × 10^6^ cells ml^−1^ in 1 liter of sterile water. Five pots containing 8 to 10 2-week-old bean plants (cv. BBL274) were inoculated by leaf immersion in the bacterial suspension for 30 s. Plants were incubated in a growth chamber at 20°C with 90% relative humidity and a 12-h light/12-h dark photoperiod. For each strain, 5 to 12 leaves were collected at each time point, and the populations were enumerated by combining four 1.3-cm-diameter leaf disks representing a leaf, homogenizing the disks in 300 µl of PB, and enumerating the cells by plating on KB medium containing Rif and Cyclo. Analysis of variance (ANOVA) was performed on the log-transformed values to evaluate differences in bacterial populations among strains at each time point, and a repeated-measures analysis of the log-transformed values was performed using a split-plot design where strain was the whole-plot factor and time was the split-plot factor, with subsampling within the split plot using Proc Glimmix in SAS.

Bacterial colonization was also examined on leaves under field conditions at the ISU Horticulture Research Farm. A randomized block design was used in which 40 bean seeds (cv. BBL274) were planted for each strain in each block of four blocks (1 by 0.5 m). Inocula contained cells that were recovered from KB medium containing 1.5% agar after a 48-h incubation, and the cells were suspended to a density of 4 × 10^7^ cells ml^−1^ in PB. Leaves of 2-week-old bean plants were inoculated by application with a hand sprayer. For each strain, four leaves were collected from each block at each time point, and bacteria were recovered by sonication for 7 min in 20 ml of washing buffer and enumerated on KB medium containing Rif and Cyclo. Differences between wild-type and mutant strains were calculated using a Student’s *t* test.
