# Supplementary figures and images for: Pseudomonas syringae pv. syringae B728a Regulates Multiple Stages of Plant Colonization via the Bacteriophytochrome BphP1
Source: mBio. 2017 Oct 24;8(5):e01178-17. doi: 10.1128/mBio.01178-17 (PMC5654926; doi:10.1128/mBio.01178-17)

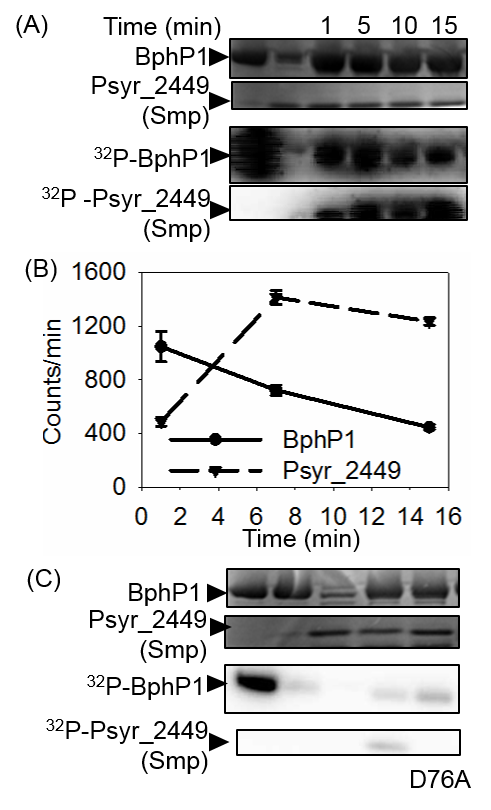

Supplement: FIG S1 [file mbo005173544sf1.tif]

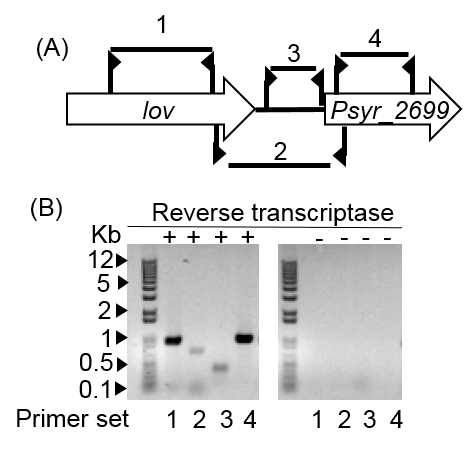

Supplement: FIG S2 [file mbo005173544sf2.tif]

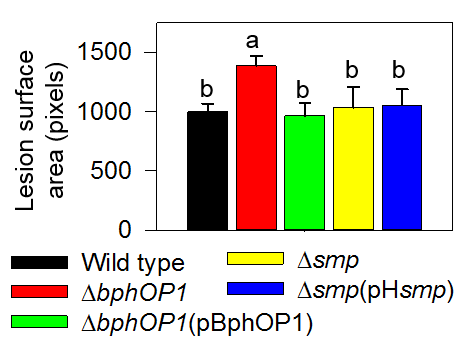

Supplement: FIG S3 [file mbo005173544sf3.tif]

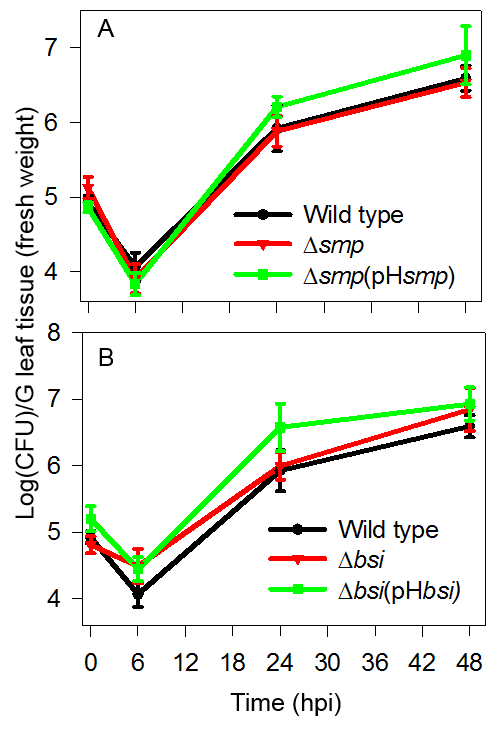

Supplement: FIG S4 [file mbo005173544sf4.tif]
