# Supplementary material for: Pseudomonas syringae pv. syringae B728a Regulates Multiple Stages of Plant Colonization via the Bacteriophytochrome BphP1
Source: mBio. 2017 Oct 24;8(5):e01178-17. doi: 10.1128/mBio.01178-17 (PMC5654926; doi:10.1128/mBio.01178-17)
Supplement: TABLE S1 [file mbo005173544st1.docx]

| **Table S1. Primers used for this study** | |
| --- | --- |
| Primer^a^ | Sequences^b^ |
| Primers for constructing deletion mutants | |
| *bsi*-FL1-F | 5’-TCGATGGTCTATGGCTTTGC-3’ |
| *bsi*-FL1-R | 5’-AGCCTACACAATCGCTCAAGACGTGCTGCATGAGTTTCGTGGTA-3’ |
| *bsi*-FL2-F | 5’-AATATCCGGGTAGGCGCAATCACTATTCTGCTGATCACGCTGGT-3’ |
| *bsi*-FL2-R | 5’-TACGATTATGTCGCCGTTGA-3’ |
| *smpR*-FL1-F | 5’-ATGCGCAGCACGGAAAAGACC-3’ |
| *smpR*-FL1-R | 5’-AGCCTACACAATCGCTCAAGACGTGGGAGGTTGCGCCAACCTGTG-3’ |
| *smpR*-FL2-F | 5’-AATATCCGGGTAGGCGCAATCACTTGGTAAATGAGCGGGCAACC-3’ |
| *smpR*-FL2-R | 5’-CTGCCGCCCTGACCCATC-3’ |
| *Kan* cassette-F | 5’-ATTGTGTAGGCTGGAGCTGCTTC-3’ |
| *Kan* cassette-R | 5’-CCATGGTCCATATGAATATCCTCC-3’ |
| Primers for expressing *bphOP1*, *bphP1*, *bphP2R*, and *lov* in the deletion mutants | |
| *bphOP1*-F | 5’-GCGGTGGCAAACCGTCGTCTTA-3’ |
| *bphOP1*-R | 5’-GCGCGAGGTACTTCCAGCGAAC-3’ |
| *bsi*-F | 5’-AAAGGACTATGACTATGATTCGCGA-3’ |
| *bsi*-R | 5’-TCAGACCGCAGCAACAACC-3’ |
| *smp*-F | 5’-CACAGGTTGGCGCAACCTCCC-3’ |
| *smp*-R | 5’-TCAAGCCAGCAGCTTCTGAA-3’ |
| Primers for site-directed mutagenesis | |
| *smp*DA-F | 5’-GATGATTACCGCTTATCGGCTGC-3’ |
| *smp*DA-R | 5’-GCAGCCGATAAGCGGTAATCATC-3’ |
| Primers for construction of His_6_-tagged proteins | |
| *bphP1*BamHIF | 5’-GCCGGATCCATGAGCCAACTCGACAAAGACGCC-3’ |
| *bphP1*NotII-R | 5’-ATTTGCGGCCGCTCAAACCGCCATTGGCACCGTGAA-3’ |
| *smp*EcoRI-F | 5’-GCGGAATTCATGCGTGCCGTGGTCGTAATGGCA-3’ |
| *smp*XhoI-R | 5’ CCCCTCGAGAGCCAGCAGCTTCTGAATCTG -3’ |
| *0886*EcoRI-F | 5’-GCGGAATTCATGTCCACGCTTGCGCTATTGATATGC-3’ |
| *0886*XhoI-R | 5’-CCCCTCGAGGCTCATCATGTAGGCGAG-3’ |
| *4376*EcoRI-F | 5’-GCGGAATTCGTGGATAACTACCCGCTCACG-3’ |
| *4376*HindIII-R | 5’-CCCAAAGCTTGCCCTGGCTTTCGCTCTG-3’ |
| *0489*EcoRI-F | 5’-GCGGAATTCATGGCTCGAATATTGATCGTC-3’ |
| *0489*XhoI-R | 5’-CCCCTCGAGGCCGGCCAGCACCGCATTCAG-3’ |
| *0488*EcoRI-F | 5’-GCGGAATTCATGGAACAACACGCCTCCGCCCTG-3’ |
| *0488*XhoI-R | 5’-CCCCTCGAGTGATATGTGTTGTTCTGCTGC-3’ |
| *3433*EcoRI-F | 5’-GCGGAATTCATGGCAGTCAAGGTCCTGGTG-3’ |
| *3433*XhoI-R | 5’-CCCCTCGAGGAGACAAGCCTCTACCAG-3’ |
| *4392*EcoRI-F | 5’-GCGGAATTCATGAGCCAGAGCCTGAGCCAG-3’ |
| *4392*XhoI-R | 5’-CCCCTCGAGCCGGTTCCCCCACAGTCG-3’ |
| *3299*EcoRI-F | 5’-GCGGAATTCATGACCTGCAATCTGTTACTGGTCGAC-3’ |
| *3299*HindIII-R | 5’-CCCAAAGCTTATCGTCCAGACTGATAATCCG-3’ |
| Primers for evaluating the *lov-bsi* operon structure | |
| *lovbsi*1-F | 5’-AAGACGGCTCCTCGTTCTGG-3’ |
| *lovbsi*1-R | 5’-AGCACCATCTTCGCCAGTTC-3’ |
| *lovbsi*2-F | 5’-CGAAGGTCAAGGTGCTGTTG-3’ |
| *lovbsi*2-R | 5’-CCAACAGGTTCAGCAACAGC-3’ |
| *lovbsi*3-F | 5’-CGATACTGACAATGAACACATGAAG-3’ |
| *lovbsi*3-R | 5’ATTGGCAGTAAACGCACACA-3’ |
| *lovbsi*4-F | 5’-ACCGTATCGCCCCAGGTG-3’ |
| *lovbsi*4-R | 5’-TCAGACCGCAGCAACAACC-3’ |
| ^a^ FL indicates flanking region  ^b^underlined portions of the primer sequence indicate the region that binds to the kanamycin cassette | |
